# Books Reviewed

**Published:** 1866-01

**Authors:** 


### Editorial Department.

### Books Reviewed.

The Student's Book of Cutaneous Medicine and Diseases of the Skin. By Erastus Wilson, F. R. S. New York: William Wood & Co; 1865.

We shall be unable in our brief space to give a better description or clearer or truer idea of the merits of this book, than will be obtained by a short quotation from the well-known author, who, in his preface, describes his object in furnishing the "*Student's Book of Cutaneous Medicine and Diseases of the Skin.*" He says: "A NATURAL CLASSIFICATION is the want of the hour; and a natural classification, if it could be attained, would, without doubt, be an important gain to our science. Alibert invented such a classification; Hardy has revived it: but we must confess that neither the classification of Alibert nor that of Hardy is such as to meet with our approval. In the present work we have framed a classification, founded on the clinical history of diseases of the skin; we have arranged these diseases into *twenty-two* groups; and we believe that for all practical purposes, the arrangement will be found sufficiently simple and comprehensive. Should it be adopted in future years as a classification worthy of being remembered, of being made the basis of study of these diseases, it may very truly be represented as a CLINICAL CLASSIFICATION.

We have not left out of view the necessity of the student being thoroughly acquainted with the skin in health, previously to undertaking the study of its diseases; and we have preceeded our chapters devoted to the twenty-two groups of diseases, by one on the Anatomy and Physiology of the Skin; and we have followed the chapter on Anatomy and Physiology, by one on the Pathology of the Skin, and the classification of its diseases. Our aim has been to simplify, to endeavor to restore to general medicine a department of much interest and importance, and, by furnishing the student with a clear view of the diseases, to remove them from the narrow sphere of specialism to the wider and nobler field of Catholic medicine."

The author has accomplished what he proposed, and consequently this book, which is comprised in near 500 pages, and elegantly published, will constitute the favorite standard not only of medical students, but of the general practitioner.

---

Stimulants and Narcotics, their Mutual Relations; with Special Researches on the Action of Alcohol, Æther and Chloroform on the Vital Organism. By Francis B. Anstie, M. D., M. R. C. P. Philadelphia: Lindsay & Blakiston; 1865.

In this book of over four hundred pages we have a most complete and well-written discussiou upon the whole range of topics embraced within the scope of stimulation and narcosis. Our author commences about the time when we first have history of the creation of the world, and gives us in a very entertaining way all the opinions which have been entertained by prominent medical men from that early period to the present time. This, as will be at once seen, comprises a history of medicine itself—of all the various doctrines which have gained ground, been adopted, and disappeared. Stimulation, irritation, vital excitation, vital action, etc., etc.—questions which have agitated the philosophical minds of every age and country—are treated with a completeness of illustration and perfection of history which renders the volume one of unsurpassed attraction to all who have time and taste for a review of the past in the theory of medicine, or the present in medical philosophy. The action and uses of stimulants and narcotics are illustrated by numerous experiments which are highly entertaining and instructive. It is withal a book full of practical suggestions, and will certainly interest and instruct the reader.

Report on the use of Pressure in the Treatment of Gonorrhœal and Purulent Ophthalmia. By Surgeon Joseph S. Hildreth, U. S.V. New York: John Medole, 1865.

Results of cases are given, and the author shows the value of pressure to be the value of an eye. If in his cases he did not use it, the eye was lost, if he did, it was saved. He describes the process of making pressure as follows:

"What I mean by the use of pressure in the treatment of such cases, is not the application of lint, wet or dry, over the lids with moderate compression, but a *firm, hard, continued pressure upon all parts of the contents of the orbit, especially the anterior.* This I effect in the following manner:

The lids being closed, the orbit is to be packed, as it were, by means of charpie, or picked lint, (scraped lint or cotton wool is not so serviceable,) in such a manner that all parts about the eye, within the orbit, the anterior hemisphere of the globe, and especially the conjunctiva, shall be acted on.

Care must be taken to fill the grand angle, and to have the charpie evenly and regularly disposed *about* as well as over the globe.

Quite a large bunch should be used for each eye, not only to ensure evenness of pressure, but to absorb the purulent discharge. This being done, compression is made by means of a bandage, or better, a firm elastic band of rubber braid, not less than two inches in width, passing around the head. It should be slowly and regularly increased until the pain, if any there be, in the parts affected, is greatly diminished or controlled, if practicable.

In other words, pressure is to be applied to the eye and surrounding parts within the margin of the orbit to a degree sufficient to so control the circulation as to prevent the destructive tendency of the disease, but not to interfere with proper nutrition. This must, of course, vary with the peculiarities of each case.

But the principle of employing, as constantly as possible, firm, hard, even and continued pressure from the *earliest moment practicable* until the *close of all acute symptoms*, is not to be lost sight of for a moment The anatomy of the orbit, the mechanism of the lids, and the cushion of adipose tissue posterior to the globe, render this not only possible, but easy.

I have in no instance resorted to it in purulent or gonorrhœal affections of the eye during the acute stages, even after the organ has been irretrievably lost, without greatly diminishing the discharge in a short time, and very materially adding to the patient's comfort in reducing the pain, and modifying subsequent and present staphyloma."

Materia Medica, for the Use of Students, by John B. Biddle, M. D.; with Illustrations. Philadelphia: Lindsay & Blakiston; 1865.

This book contains a succinct account of all the articles of materia medica, and is designed as a text-book to lectures upon this branch in our medical colleges. It is well illustrated by representations of the most important indigenous and naturalized plants used in medicine, which adds greatly to its value as a text-book for medical students. It is sufficiently copious and complete to be of value to the general practitioner who has time for brief reference, and desires to obtain only important practical facts.

Specialties in Medicine. By Henry D. Noyes, M. D. Read before the American Ophthalmological Society, June, 1865.

This is a very carefully and fairly written argument in favor of specialists; and the positions are so well taken and so fully sustained as to do much towards removing all prejudice in the minds of general practitioners. If specialists were really what Dr. Noyes represents they should be, and behaved themselves as he says they ought to behave, there could be no objection urged against them, on the other hand everything would be in their favor. We commend this address with great earnestness to both the specialist and general physician; the former will learn what he ought to be and do, and the latter how he should regard and treat the true specialist.

